# BLOOD PRESSURE TRENDS PRECEDING DEMENTIA: A STUDY OF HEART FAILURE PATIENTS

**DOI:** 10.1093/geroni/igz038.3478

**Published:** 2019-11-08

**Authors:** Connor Wilson, Amanda Pangle, Jeanne Wei, Gohar Azhar

**Affiliations:** Donald W. Reynolds Institute on Aging, UAMS, Little Rock, Arkansas, United States

## Abstract

Blood pressure and perfusion of the brain are central components of neurological health that are often influenced by heart failure. This retrospective case-control study analyzed blood pressure changes preceding the diagnosis of dementia in patients over the age of 60. Blood pressures were obtained from the date of dementia diagnosis, and then one year and five years before diagnosis. Study “controls” were age-matched patients without dementia, using the mean age of dementia diagnosis as the first data point. Over the five-year period preceding diagnosis, 67.2% with dementia showed decreasing systolic pressure compared to 46.6% of patients without dementia. Similarly, 62.7% with dementia showed a decreasing systolic pressure over the one-year period, compared to 50.0% of those without dementia. Additionally, there was a significant difference (p < 0.001) in the dementia rates between African American and Caucasian subgroups (55.0% African Americans vs. 31.0% Caucasians). Patients with dementia were more likely to have decreasing blood pressure trends than age-matched patients without dementia and appeared to have significantly lower blood pressures one year before the diagnosis. It is crucial that providers are cognizant of these trends and risk factors for dementia as they manage blood pressures in geriatric patients.

